# Towards a better preclinical cancer model – human immune aging in humanized mice

**DOI:** 10.1186/s12979-023-00374-4

**Published:** 2023-09-27

**Authors:** Joel H. L. Tan, You Yi Hwang, Hui Xian Chin, Min Liu, Sue Yee Tan, Qingfeng Chen

**Affiliations:** 1 Institute of Molecular and Cell Biology (IMCB), Agency for Science, Technology and Research (A*STAR), 61 Biopolis Drive, Proteos, Singapore, 138673 Republic of Singapore; 2 Singapore Immunology Network (SIgN), Agency for Science, Technology and Research (A*STAR), 8A Biomedical Grove, Immunos Level 3 & 4, Singapore, 138648 Republic of Singapore; 3 Department of Microbiology and Immunology, Yong Loo Lin School of Medicine, National University of Singapore, Singapore, Republic of Singapore

**Keywords:** Humanized mice, Immune aging, T cells, Cancer

## Abstract

**Background:**

Preclinical models are often used for cancer studies and evaluation of novel therapeutics. The relevance of these models has vastly improved with mice bearing a human immune system, especially in the context of immunotherapy. Nonetheless, cancer is an age-related disease, and studies often overlook the effects of aging. Here we have established a humanized mouse model of human immune aging to investigate the role of this phenomenon on liver tumor dynamics.

**Methods:**

Multiple organs and tissues (blood, thymus, lung, liver, spleen and bone marrow) were harvested from NOD-*scid IL2rγ*^*−/−*^ (NIKO) mice reconstituted with human immune cells, over a period of 60 weeks post-birth, for immune profiling. Young and aging immune cells were compared for transcriptomic changes and functional differences. Effect of immune aging was investigated in a liver cancer humanized mouse model.

**Results:**

Focusing on the T cell population, which is central to cancer immunosurveillance and immunotherapy, we showed that the proportion of naïve T cells declined while memory subsets and senescent-like cells increased with age. RNA-sequencing revealed that downregulated genes were related to immune responses and processes, and this was corroborated by reduced cytokine production in aging T cells. Finally, we showed faster liver tumor growth in aging than younger humanized mice, which could be attributed to specific pathways of aging T cell exhaustion.

**Conclusion:**

Our work improves on existing humanized (immune) mouse model and highlights the importance of considering immune aging in liver cancer modeling.

**Supplementary Information:**

The online version contains supplementary material available at 10.1186/s12979-023-00374-4.

## Background

Aging is an inevitable phenomenon that affects everyone. As a population ages, the frequency of chronic, infectious, and autoimmune diseases, which are often correlated with an aging immune system, increases [1]. To better manage these age-related diseases, it is pertinent to understand human immune aging. Aging research in humans faces many challenges, such as our long life expectancy, which makes it resource demanding, and the limitations on biological sampling and testing [2]. The mouse has been a choice model for many areas of in vivo biological research, including aging, because of its ease of generation and relatively short lifespan of up to three years [2]. Mice also share many age-related phenotypes found in humans and have been used to demonstrate that the aging process can be delayed, for example through caloric restriction [3, 4]. Interestingly, the life-extending effect of caloric restriction was associated with a reduction in expression of inflammatory genes [5, 6], highlighting the important role the immune system plays in aging. Research in mice has revealed nine molecular hallmarks of mammalian aging: genomic instability, telomere attrition, epigenetic alterations, loss of proteostasis, deregulated nutrient sensing, mitochondrial dysfunction, cellular senescence, stem cell exhaustion, and altered intercellular communication [7]. These hallmarks contribute to an imbalance in homeostasis, as well as a dysfunctional immune system, which could lead to multiple age-related diseases such as cancer, arthritis, diabetes, and cardiovascular complications [8–11]. Because a properly functioning immune system is vital for recognizing and killing tumor cells [12], immunosenescence caused by aging is a risk factor for cancer and a negative indicator for immunotherapy efficacy [13].

In spite of the knowledge obtained from studying mouse models, the applicability of mouse physiology to human biology remains in question [14, 15]. For example, mice and humans have opposite circadian rhythms, and our laboratory has shown that this is consistent in circulating leukocytes, owing to the divergent expression of C-X-C chemokine receptor 4 [16]. Moreover, there are many instances in drug development where responses in mice differed significantly from those in humans, contributing to the high dropout rate of candidate drugs that enter clinical trials [17, 18]. To address this issue, mouse models need to better recapitulate human biology, at least in a manner that is relevant to the system or disease under investigation. For instance, engrafting human hepatocytes in the liver of immunocompromised mice has enabled the model to more accurately predict human drug metabolism and response [19]. In another example, HIV research using mouse as a model requires the reconstitution of a human immune system in mice to overcome the barrier of viral tropism [20].

In this study, we used immunocompromised mice (NOD-*scid IL2rγ*^*−/−*^*,* NIKO mice) reconstituted with a functional human immune system (through engraftment of human CD34^+^ hematopoietic stem cells) to comprehensively determine the temporal changes in human immune cells in various tissues (blood, thymus, lung, liver, spleen, and bone marrow). Since T cells are instrumental against cancer and have become a major target for cancer immunotherapy [21], we focused on T cells in humanized mice and explored the role of these aging cells in human tumor development.

## Results

### Reconstitution of human immune cells in NIKO mice

**Fig. 1:**
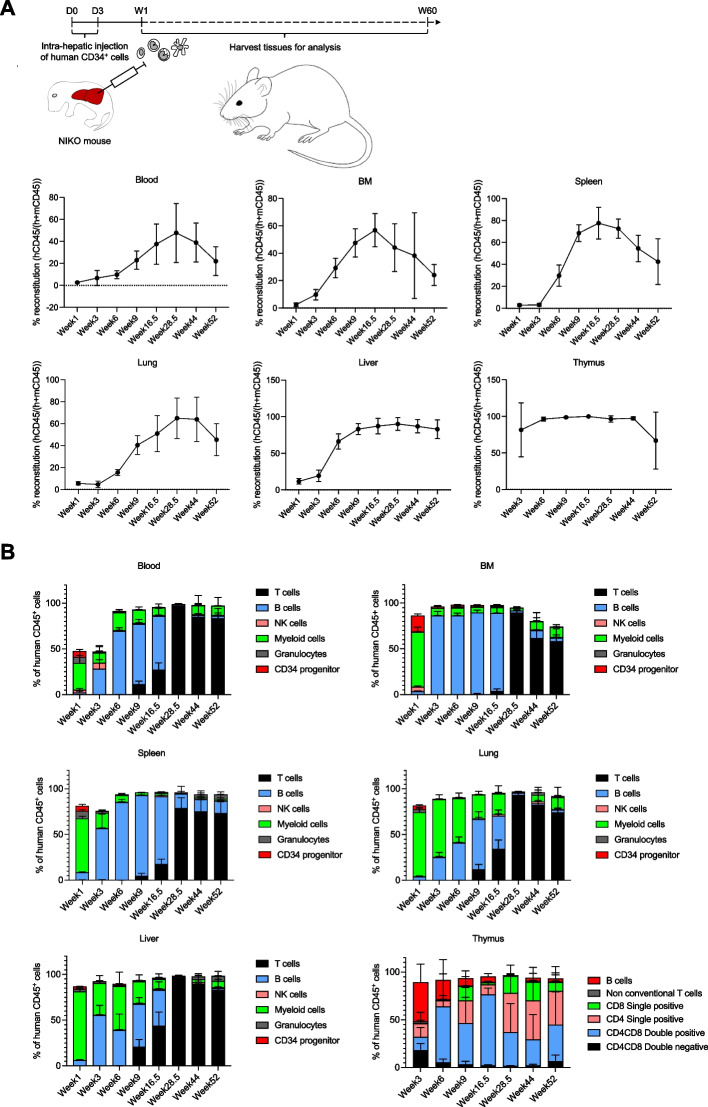
Human immune cell reconstitution of NIKO mice. **A** Timeline for engrafting human CD34^+^ cells and harvesting of blood and organs for analysis. Percentage reconstitution of human immune cells in blood, bone marrow, spleen, lung, liver and thymus of NIKO mice over a course of 52 weeks. mean ± SD are indicated; *n* = 5. **B** Proportion of various human immune subsets in the blood, bone marrow, spleen, lung and liver over time. For the thymus, immune cells largely comprised of B and T cells, proportion of subsets as shown. Bars show mean ± SD; *n* = 5

Neonate NIKO mice were engrafted with human CD34^+^ cells via intrahepatic injection. Starting from one-week post-partum, peripheral blood, thymus, lung, liver, spleen and bone marrow were harvested to analyze the reconstitution of human immune cells in these organs. We observed successful human immune cell reconstitution across all organs of the NIKO mouse, especially the vestigial thymus. Most tissues analyzed saw increased relative percentages of human CD45^+^ versus combined human and mouse CD45^+^ cells over time, peaking at 16.5 to 28.5 weeks post-partum. The degree of reconstitution varied amongst organs, but at the peak, human CD45^+^ immune cells comprised greater than 50% of all CD45^+^ cells in all organs. The thymus was the exception, which displayed near complete reconstitution of human immune cells from 6 weeks post-partum. As the mice aged, the relative percentage of human CD45^+^ cells declined in all organs from its peak (Fig. 1A, Additional file 1: Fig. S1), possibly due to reduced proliferation of these cells or increased proliferation of mouse immune (myeloid) cells.

We immunophenotyped the human CD45^+^ cells to track changes in the human immune cell population over 52 weeks and observed drastic changes in the composition of leukocytes in all organs. One week after human CD34^+^ cell engraftment, all tissues (excluding the thymus) were dominated by a combination of HLA-DR^+^ myeloid cells, residual CD34^+^ progenitor cells, and CD66b^+^ granulocytes (Fig. 1B, Additional file 1: Fig. S1). B cells were the next primary cell type to appear during week 3 and dominated the hematopoietic compartment up to week 16.5, while the myeloid population correspondingly decreased. This was especially true in the blood, spleen and bone marrow, where B cells comprised the majority of immune cells from week 3 to 16.5. The liver and lung appeared to support the myeloid cell population the longest, comprising 23.73% and 22.59% of human CD45^+^ cells in the liver (up to week 9) and lung (up to week 16.5), respectively. T cells began to appear in the blood, spleen, lung and liver at week 9, and in the bone marrow at week 16.5. They quickly surpassed all other cell types, making up the majority of the human CD45^+^ cells by week 28.5 in the spleen (78.87%), bone marrow (89.44%), lung (93.42%), liver (96.67%) and blood (97.06%) (Fig. 1B, Additional file 1: Fig. S1).

The thymus showed a skew towards the adaptive immune cell compartment from week 3, being dominated by B cells (41.02%), single positive CD4 (13.94%) T cells, as well as the expected CD3-positive, CD4/CD8 double positive (DP) (13.97%) and double negative (DN) (18.13%) thymocytes. The frequency of B cells steadily decreased as the mice matured. This was accompanied by an increase in DP T cells, peaking at week 16.5 (74.05%) and decreasing with age, thereafter, denoting appropriate thymopoiesis of human T cells (Fig. 1B, Additional file 1: Fig. S1).

Overall, human CD34^+^ cells, when transplanted into the liver of neonate NIKO mice, can reconstitute various human immune cell types, the composition of which drastically changes as the mice age. While there was a good mixture of T, B and myeloid cells up to week 16.5, the mice exhibited a T cell-dominant phenotype from week 28.5 onwards. This phenomenon was consistent across all organs except for the thymus, which led us to focus our study on T cells.

### Population shifts of T cells in aging humanized mice

**Fig. 2:**
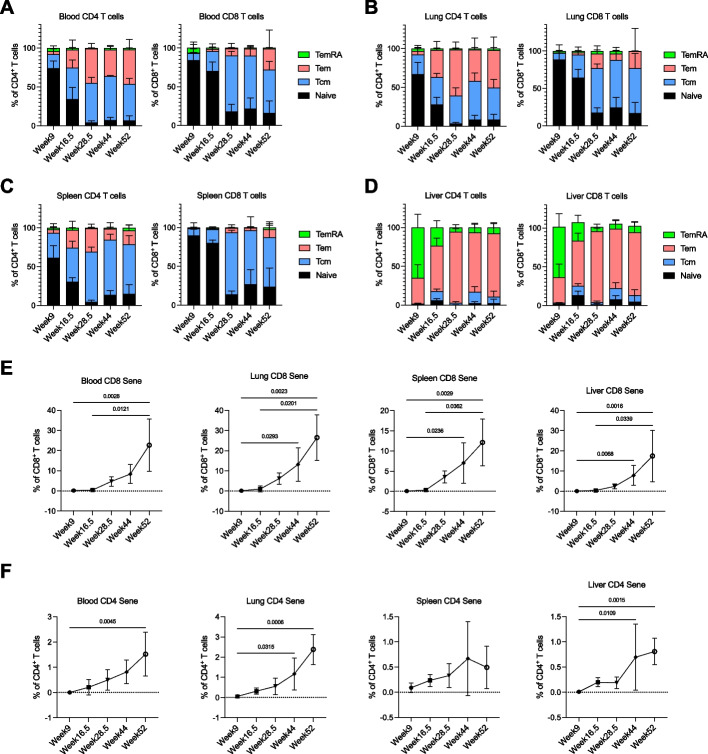
Changes in T cell population as humanized mice aged. **A** to **D** Proportion of naïve and memory CD4^+^ and CD8^+^ T cell subsets in blood (**A**), lung (**B**), spleen (**C**) and liver (**D**). Bars show mean ± SD; *n* = 5. **E** and **F** Percentage of CD8^+^ T cells (**E**) and CD4.^+^ T cells (**F**) that express senescent makers, CD57 and KLRG1, over time. Kruskal–Wallis test was used for significance tests. mean ± SD and significant *p* values are indicated; *n* = 5

One of the effects that aging has on the human T cell population is the decreased proportion of naïve T cells in the blood, with a corresponding increase of memory and senescent T cells [22]. In humanized mice, we found that in the blood, lung and spleen, there was a significant decrease in the proportion of naïve CD4^+^ and CD8^+^ T cells starting after weeks 9 and 16.5, respectively, to week 52 (Fig. 2A-C, Additional file 2: Fig. S2A-C). This decrease was accompanied by a concomitant increase in memory subsets (Fig. 2A-C). However, this phenomenon was not apparent in the liver, likely due to the inherently low proportion of naïve cells even in younger mice (Fig. 2D, Additional file 2: Fig. S2D), which could be attributed to the activation of naïve cells through antigen presentation by liver-resident cells [23]. The declining naïve and increasing memory T cell compartments in aging humans were hence mirrored in the aging humanized mice.

As cellular senescence is one hallmark of aging, we investigated whether the T cells also displayed increasing surface markers of cellular senescence with age. Defining senescent-like T cells as KLRG1^+^CD57^+^ cells [24–26], we observed that in blood, lung, spleen and liver, there was a progressive increase in the proportion of senescent-like CD8^+^ T cells over time from week 9 to 52. At week 52, the proportion of senescent-like cells was significantly higher than week 9 with 12.11%, 17.44%, 22.67% and 26.50% in the spleen, liver, blood, and lung, respectively (Fig. 2E). A similar phenomenon was seen, albeit to a lesser extent, for the CD4^+^ T cells in the blood, lung and liver, but not the spleen (Fig. 2F). As such, our humanized mouse model recapitulated T cell population changes in aging humans.

### Population and functional insights through single cell RNA sequencing

**Fig. 3:**
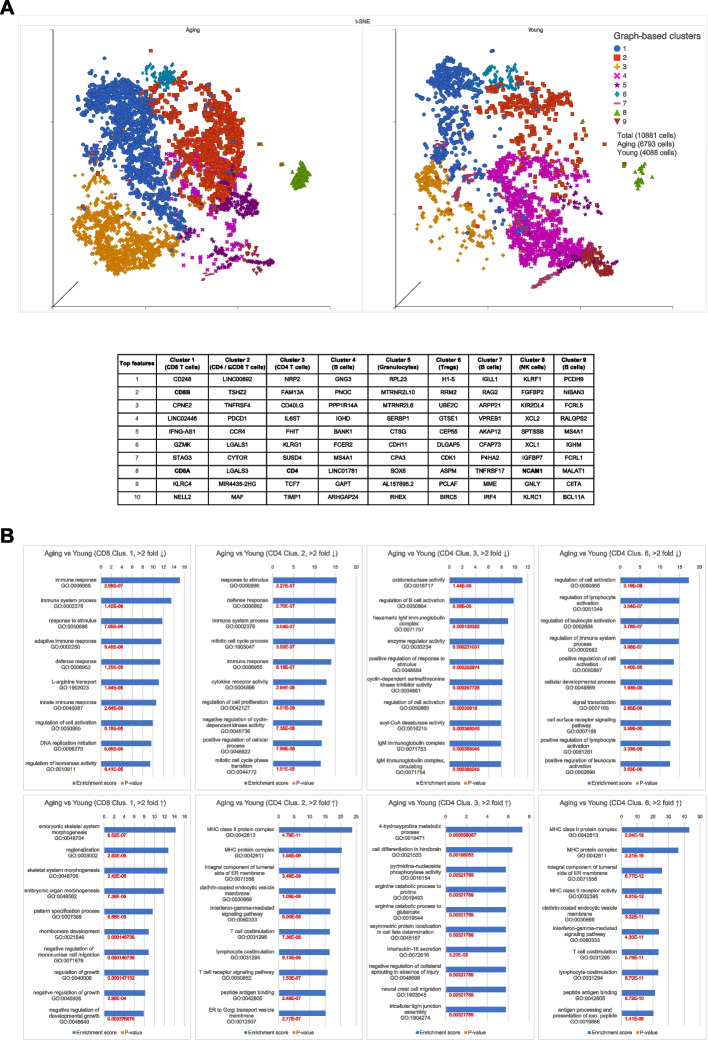
Single cell RNA sequencing analysis of splenocytes. **A** tSNE plot showing nine graph-based clusters that potentially represent different human immune subsets found in splenocytes harvested from aging (55-week-old) and young (14-week-old) humanized mice. Table shows top ten expressed genes for each cluster with likely cell type in parenthesis. **B** Gene set enrichment analysis of genes that were up- or down-regulated by at least twofold. Top ten enriched GOs are shown for CD8^+^ cells in Cluster 1, CD4^+^ cells in Cluster 2, CD4^+^ cells in Cluster 3 and CD4^+^ cells in Cluster 6. *p* values are indicated

To better understand the population shifts between young and aging immune cells, we performed single cell RNA-sequencing on splenocytes from humanized mice, as the spleen has been assessed to be a good indicator of systemic immune functions [27]. After quality control and filtering, a total of 10,881 (4,088 young and 6,793 aging) immune cells were analyzed. The data was visualized by t-distributed stochastic neighbor embedding (tSNE), and the cells were grouped into nine clusters. Combining information on the top ten features with specific marker genes (CD3, CD4, CD8, CD19 and NCAM1), the clusters potentially represent (to a large extent) CD8^+^ T cells (Cluster 1 and subset of 2), CD4^+^ T cells (Clusters 2 and 3), B cells (Clusters 4, 7 and 9), Granulocytes (Cluster 5), Regulatory T (Treg) cells (Cluster 6), and Natural Killer (NK) cells (Cluster 8) (Fig. 3A, Additional file 4: Fig. S4). Consistent with our earlier findings, aging splenocytes largely comprised of T cells, with a much smaller B cell population compared to young splenocytes. A closer look at the tSNE plot revealed that T cells from aging mice within T cell clusters 1, 2 and 3 were situated lower than those from younger mice along the vertical tSNE plane, indicating a distinct shift in CD8^+^ and CD4^+^ T cell transcriptome with age (Fig. 3A, Additional file 4: Fig. S4). Unexpectedly, *KLRG1* expression in these T cells did not appear to correlate with age (Additional file 4: Fig. S4).

Since this study focuses on T cells (CD3^+^), we compared the differentially expressed genes between aging and young CD3^+^ cells within CD8^+^ Cluster 1, CD4^+^ Cluster 2, CD4^+^ Cluster 3, and CD4^+^ Cluster 6 (Tregs). Genes that were significantly up- and down-regulated by more than two-fold were subjected to Gene Set Enrichment Analysis (GSEA) to yield Gene Ontologies (GO) that provide insights into affected molecular functions and biological processes. Looking at CD8^+^ T cells of Cluster 1, genes that were significantly downregulated in aging cells are associated with immune responses (adaptive and innate), response to stimulus, immune system process and defense response, while those that were significantly upregulated are related to developmental processes such as morphogenesis (Fig. 3B, far left). Aging CD4^+^ T cells in Cluster 2 also expressed lower levels of genes that correspond to immune processes, as well as mitosis and cell proliferation. These cells instead showed increased expression of genes involved in antigen presentation and T cell stimulation (Fig. 3B, left). For their counterparts in Cluster 3, downregulated genes corresponded to oxidoreductase activity, regulation of B cell activation, and IgM immunoglobulin complex, while upregulated genes were enriched in amino acid metabolism and nucleoside phosphorylase activity (Fig. 3B, right). Finally, for Tregs in Cluster 6, the downregulated genes were associated with activation of immune cells and regulation of immune system, while the upregulated genes were associated with MHC Class II processes and lymphocyte co-stimulation (Fig. 3B, far right). Taken together, single cell RNA-sequencing of aging and young splenocytes revealed immune population shifts as well as changes in molecular functions and biological processes that could explain age-related morbidity, such as increased tumor incidence and progression.

### T cell dysfunctions in aging humanized mice

**Fig. 4:**
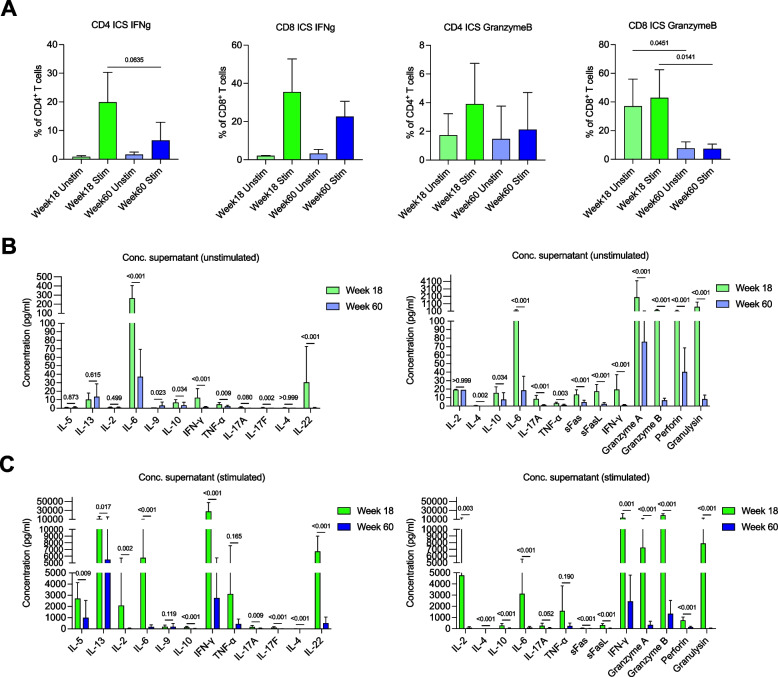
In vitro analysis of T cell function (**A**) Intracellular staining of splenocytes for interferon-gamma and granzyme B, 4 h after culture with or without PMA/ionomycin stimulation. CD4 and CD8 cells were distinguished by FACS. Bars show mean ± SD and *p* values are indicated; *n* = 5. **B** and **C** Analysis of cytokines in the supernatant 72 h after culturing splenocytes in the absence (**B**) or presence (**C**) of anti-CD3/28 stimulation, using LEGENDplex™ T helper cytokine panel (left) or LEGENDplex™ CD8/NK panel (right) to probe for T helper or CD8/NK related cytokines, respectively. Bars show mean ± SD and *p* values are indicated; *n* = 5. Mann–Whitney test was used for all significance tests

Given the reduced proportion of naïve and increased proportion of senescent-like T cells in aging humanized mice, as well as the GOs affected by transcriptomic changes, we next investigated the functional aspects of the aging T cells. We performed in vitro stimulation, using PMA and ionomycin, and intracellular staining (ICS) of splenocytes from week 18 and week 60 humanized mice to compare the intrinsic capability of T cells to produce cytokines. The proportion of IFNγ-producing CD4^+^ and CD8^+^ T cells at week 60 was lower compared to week 18, even though it was not significant. Strikingly, there was a significant reduction in the proportion of Granzyme B positive CD8^+^ T cells at week 60 compared to week 18, regardless of in vitro stimulation. Similarly, the proportion of Granzyme B positive week 60 CD4^+^ T cells was about half that of their week 18 counterparts, although the difference was not significant (Fig. 4A).

In addition to looking at intrinsic cytokine production capability of the cells, we simulated T cell receptor-dependent activation using anti-CD3 and anti-CD28 antibodies in vitro for 72 h. The cytokine profile of the culture supernatant was measured using BioLegend’s LEGENDplex™, and the results supported the ICS data, where week 60 splenocytes produced significantly less IFNγ and Granzyme B compared to week 18 splenocytes. The bead-based immunoassay also showed that while week 18 T cells were functionally capable of producing a wide range of pro-inflammatory and cytotoxic cytokines such as IL-5, IL-13, IL-6, IFNγ, TNF-α, IL-17A, IL-22, GranzymeA, GranzymeB, Perforin, and Granulysin in response to physiological activation, week 60 splenocytes were largely deficient in this regard (Fig. 4B, C). Since our results demonstrated that aging humanized mice were dominated by T cells throughout multiple organs, and that these T cells were functionally compromised compared to their younger counterparts, we theorized that aging mice would have impaired tumor-killing capacity.

### Disparity in tumor dynamics between aging and young humanized mice

**Fig. 5:**
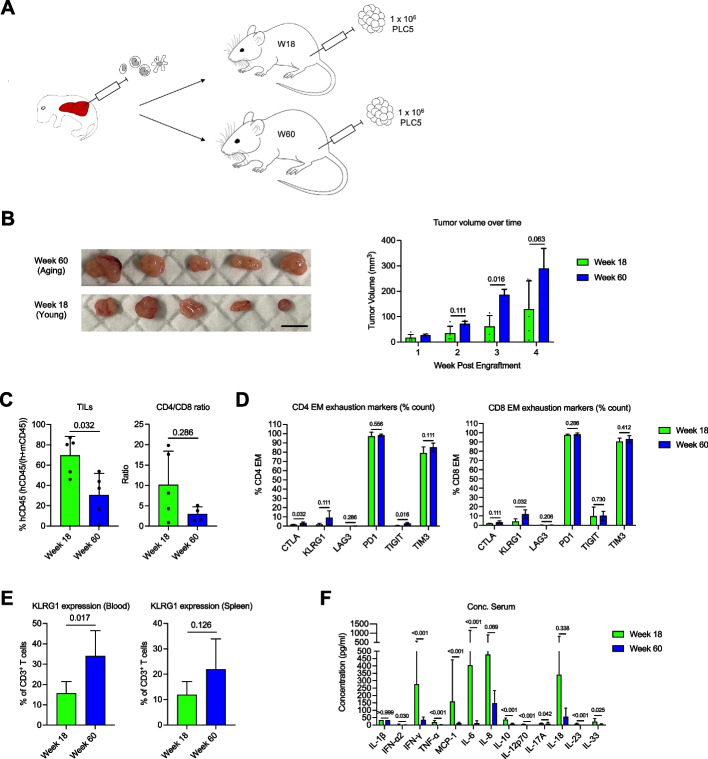
Tumor study in aging and young humanized mice. **A** Schematic diagram showing reconstitution of NIKO with human immune cells and subsequent engraftment of liver cancer cell line PLC/PRF/5 in young (Week 18) and aging (Week 60) humanized mice. **B** Pictures of tumors harvested 4 weeks post engraftment. Scale bar, 1 cm. Change in tumor volume over time. Bars show mean ± SD and *p* values are indicated; *n* = 5. **C** Analysis of TILs for percentage of hCD45 and CD4/CD8 ratio. Bars show mean ± SD and *p* values are indicated; *n* = 5. **D** Analysis of TILs for percentage of cells expressing exhaustion markers. Bars show mean ± SD and *p* values are indicated; *n* = 5. **E** KLRG1 expression on CD3^+^ T cells in blood and spleen of non-tumor engrafted humanized mice. Bars show mean ± SD and *p* values are indicated; *n* = 5. **F** LEGENDplex™ analysis of serum from tumor engrafted mice using Inflammation panel 1, 4 weeks post engraftment. Bars show mean ± SD and *p* values are indicated; *n* = 5. Mann–Whitney test was used for all significance tests

We examined the effects of immune aging on tumor dynamics in our humanized mouse model. Human hepatocellular carcinoma cells (PLC/PRF/5) were subcutaneously engrafted into the flanks of aging (week 60) and young (week 18) humanized mice, which were then monitored for tumor growth over 4 weeks (Fig. 5A). Tumors harvested from aging mice were comparatively bigger than those from young mice (288.7mm^3^ vs 129.1mm^3^) at 4 weeks (Fig. 5B), an observation that aligns with the higher incidence of cancer in the aging human population. Looking at tumor volume over time, aging mice had consistently larger tumors that were approximately up to three times the size of the tumors from their younger counterparts (Week 1, 27.2 mm^3^ vs 16.8 mm^3^; Week 2, 72.2 mm^3^ vs 34.4 mm^3^; Week 3, 185.9 mm^3^ vs 61.5 mm^3^) (Fig. 5B).

To understand the interplay between T cells and tumor development, we isolated the TILs, which largely comprised of CD3^+^ T cells, for flow cytometric analysis. Strikingly, the percentage of hCD45 in young mice (69.7%) was significantly higher than aging mice (30.4%) (Fig. 5C, left). Aging mice also presented with a lower proportion of CD4^+^ and a higher proportion of CD8^+^ T cells compared to young mice, and thus a lower CD4/CD8 ratio, although this was not significant (Fig. 5C, right). As T cell exhaustion is a common phenomenon related to reduced anti-tumor response [28], we looked at exhaustion markers. Inhibitory receptors programmed cell death protein 1 (PD-1) and T-cell immunoglobin domain and mucin domain protein 3 (TIM3) were highly expressed in similar proportions of aging and young effector memory T cells, reflecting a typical exhausted phenotype of TILs, and suggesting that these pathways are not responsible for the difference in tumor development. The exhaustion markers cytotoxic T-lymphocyte-associated protein 4 (CTLA-4, 3.05% vs 1.47%) and T cell immunoreceptor with Ig and ITIM domains (TIGIT, 2.96% vs 0.54%) were expressed in a significantly higher proportion of aging CD4^+^ effector memory T cells (Fig. 5D, left), while KLRG1 (11.88% vs 3.95%) was expressed in a significantly higher proportion of aging CD8^+^ effector memory T cells (Fig. 5D, right). CTLA-4, TIGIT and KLRG1 may thus contribute to the reduced anti-tumor activity in aging mice. In fact, KLRG1 expression on CD3^+^ T cells was already elevated in the blood and spleen of age-matched aging humanized mice in the absence of tumor (Fig. 5E). This T cell exhaustion phenotype was corroborated by the significantly lower concentrations of various T-cell associated inflammatory cytokines, such as IFN-γ, TNF-α, IL-6 and IL-17A, in the plasma of tumor-bearing aging mice (Fig. 5F). Taken together, these data suggest that the exhaustion of aging T cells could account for the more aggressive tumor dynamics in aging mice.

## Discussion

Their relatively short lifespan and ability to support a human immune system make humanized mice a practical and relevant model to study human immune aging as well as aging-related diseases. Although human CD34^+^ cells, when transplanted into the liver of neonate NIKO mice**,** were able to differentiate into various human immune cells, reconstitution of myeloid subsets was generally poor and T cells tend to emerge as the dominant subset over time, as previously reported (at least in the peripheral blood) [29, 30]. Interestingly, in the liver and lung, we see better reconstitution of human myeloid cells in adult humanized mice, possibly due to the development of resident cells from hematopoietic stem cells (HSC) and their ability to self-maintain by local proliferation [31]. To address the general paucity of myeloid subsets, depending on the disease model and immune subset of interest, cytokines like granulocyte–macrophage colony-stimulating factor (GM-CSF), macrophage colony-stimulating factor (M-CSF) and Interleukin 15 can be administered to improve the reconstitution of certain populations [32]. A major drawback of using CD34^+^ HSC reconstituted humanized mice appears to be the inter-animal variability in reconstitution levels. To address this, a longitudinal analysis approach (monitoring the same mice over a period) could be adopted. In an earlier study, longitudinal analysis of B cells in peripheral blood saw a gradual decrease in levels at 16 weeks (49.5%), 24 weeks (24.6%) and 32 weeks (18.8%) post humanization, but this trend was considerably different when multiple groups of mice were used – 16 weeks (63.7%), 24 weeks (27.7%) and 32 weeks (55.6%) [30]. While more variability and trend anomalies can be expected when multiple groups of mice are used for different time points, this study is important to track reconstitution of human immune cells in various organs over time, which requires end-point euthanasia for each time point.

Since T cells are central to cancer immunosurveillance and immunotherapy [21], we focused on the temporal development of these cells in humanized mice. Similar to humans, immune aging in our humanized mouse model involves a decrease in peripheral naïve T cells and increase in memory subsets [33]. While the decrease in naïve (and increase in memory) T cells may be attributed to thymic involution and latent persistent infections [34], homeostatic proliferation of prevailing naïve T cells [35] and the relatively clean housing conditions of the mice, respectively, suggest that there might be T cell intrinsic factors involved, or that the aging host microenvironment influences their differentiation. We noted that there is a humanized mouse model of human thymus engraftment in addition to CD34^+^ HSC but monitoring was only done up to 22 weeks post engraftment and immunophenotyping mainly on CD4^+^ cells, likely due to the study’s focus on thymic involution [36]. It would be interesting to include the engraftment of human thymus along with the transfer of CD34^+^ HSC in the context of our study, but this will be hindered by sample availability (matched human thymic tissue and CD34^+^ cells) and technical challenge of thymic engraftment in mouse pups. In our study, we showed that T cells became more senescent-like with age, as defined by increased surface expression of the senescence markers CD57 and KLRG1. Although it has been reported that T cell senescence is associated with elevated production of pro-inflammatory cytokines such as IL-6 and IFN-γ [37], we observed a general decrease in the secretion of T helper- and CD8/NK-related cytokines by aging cells (especially with anti-CD3 and anti-CD28 stimulation), suggesting that exhaustion could be preventing T cell activation.

Consistent with humans, where age is positively correlated with tumor progression [38], aging humanized mice showed better tumor growth. Upon analyzing the TILs, we observed a lower CD4/CD8 ratio in aging mice, which can be associated with immunosenescence [39, 40] as well as poorer disease outcome for certain cancers [41, 42]. Inhibitory receptors such as PD-1 and TIM3 were highly expressed in aging and young effector memory T cells, indicating an exhaustion phenotype typical of TILs [28] but excluding their involvement in age-related tumor progression. Of interest is the senescent marker, KLRG1, which is also a co-inhibitory receptor that regulates the activity of T and NK cells. As PLC/PRF/5 cells express E- and N- Cadherins [43], ligands of KLRG1, the elevated levels of KLRG1 in the effector memory cells of aging mice might have contributed to greater inhibition of anti-tumor activity [44]. This was reflected in the lower plasma levels of the T cell-associated inflammatory cytokines IFN-γ, TNF-α, IL-6 and IL-17A. While we cannot rule out the possibility that the lower level of these inflammatory cytokines could be attributed to the gradual loss of myeloid cells that can influence T cell activity [45], as evidenced by the significantly lower level of myeloid-related cytokines (IL-8, IL-18 and MCP-1), our data showed that intrinsic T cell function was certainly impacted by aging. Consequently, the low percentage of human TILs in aging mice might be contributed by reduced homing (to tumor) and proliferation of exhausted or senescent T cells.

Finally, single cell RNA-sequencing of aging and young splenocytes revealed population shifts in T cell subsets. Transcriptomic analysis comparing these subsets provided insights on changes in immune functions that could be related back to the tumor phenotype. These changes include the downregulation of genes that are implicated in the activation, ability to respond to stimulus and immune response of cytotoxic CD8^+^ T cells, indicating impaired killing of neoplastic cells [46]. CD4^+^ T cells that mediate anti-tumor immunity through their secretion of effector cytokines, support of CD8^+^ T cell functions, and coordination of antibody responses underwent changes in gene expression that affected their response to stimulus, immune response, proliferation, and regulation of B cells [47]. Aging CD4^+^ Tregs also contributed to lower levels of T cell activation. These GOs of downregulated genes in CD8^+^ and CD4^+^ T cells were consistent with the cytokine data. Interestingly, GOs associated with upregulated genes in CD8 T cells were mainly related to embryonic development. Such reversion in the aging transcriptome suggests a deregulation of gene expression networks potentially caused by accumulation of damages at the genetic or epigenetic level [48]. Unexpectedly, increased gene expression in aging CD4^+^ T cells and Tregs was linked to T cell stimulation/activation GOs – MHC class II complex [49, 50], T cell and lymphocyte co-stimulation, and T cell receptor signalling pathway – implying a positive feedback loop to stimulate anergic CD4^+^ T cells. While transcriptomic data provides insights on the state and function of the cells, it is best coupled with proteomic data for a comprehensive analysis of biological processes, especially in dynamic conditions such as age-dependent cell differentiation, since transcript level does not always accurately predict protein expression [51].

## Conclusions

In conclusion, humanized mouse models are a practical option to study human immune aging, especially in T cells. Since T cell aging does affect tumor progression, it is important that researchers consider this aspect when designing their assays to evaluate novel therapeutics.

## Methods

### Generation of humanized mice

All animal work was conducted in accordance with the A*STAR approved IACUC protocols (#221,686 and #221,738). NOD-*scid IL2rγ*^*−/−*^ (NIKO) mice were generated by A*STAR Biological Resource Centre and bred under conditions free of specific pathogens with a 12 h light–dark cycle. NIKO mice are similar to NSG mice in that they are of the same NOD strain with *Prkdc*^*scid*^ and *IL2rγ* mutations that render them severely immunocompromised – absence of mature T and B cells, lack of functional NK cells and deficiency in cytokine signalling. Our laboratory has found no functional/phenotypic difference between NIKO and NSG in our experiments. One to three-day-old NIKO pups were exposed to 1 Gy of radiation prior to intrahepatic injection of 2 × 10^5^ CD34^+^ human hematopoietic stem cells (STEMCELL Technologies). Neither male nor female was specifically selected for in this study. Mice that exhibited any sign of Graft-versus-host-disease (e.g. alopecia) were excluded from the analysis.

### Tissue harvesting and dissociation

Five unidentified mice were randomly selected (per time point), from a pool of humanized mice generated using the same batch of CD34^+^ cells and euthanized with carbon dioxide. Blood was collected by cardiac puncture into a 0.5 mL EDTA blood tube. Perfusion was performed before organ harvesting in the following order: Spleen, liver, thymus, lung, and bone marrow. All the organs were placed in 1 mL of PBS except bone marrow. For liver and lung tissues, a single-cell suspension was generated by incubating in modified RPMI (Gibco) supplemented with 10% heat-inactivated FBS (Gibco), 20 µg/mL DNAse I (Sigma-Aldrich) and 200 µg/mL Collagenase (Sigma-Aldrich). The samples were mechanically diced and incubated for 1 h at 37 °C. These samples along with lymph node, spleen and thymus were dissociated into single cells by passing through a 100 µm cell strainer. FACS Buffer (PBS with 5% FBS) was used to flush out bone marrow cells from the cavities. Red blood cell (RBC) lysis buffer was added to blood and single cell suspension containing RBC. The cells were then counted and assessed for viability with Trypan Blue (Sigma-Aldrich).

### Immunophenotyping of leukocytes

All cells except those from the bone marrow were stained with the following anti-human antibodies: CD25 (BB515), CD127 (BB700), CD3 (BUV395), CD56 (BUV563), HLADR (BUV661), CD27 (BUV737), CD33 (BUV737), CD45 (BUV805), CD57 (AlexaFluor647), CD123 (AlexaFluor647), CD19 (APC-R700), PanGDT (APCR700), CD14 (APCCy7), CD161 (APCCy7), CCR7 (PE), KLRG1 (PEDazzle594), CD11c (PeCy5), FceR1a (PeCy7), Vd1 (PE-Vio 770), CD66b (BV421), CD62L (BV480), CD16 (BV510), CD20 (BV510), CD8 (BV570), IgM (BV570), CD38 (BV605), CD45Ra (BV650), IgD (BV711), Vd2 (BV711), CD4 (BV750), CD34 (BV786), Va7.2 (BV786). Cells isolated from bone marrow were stained with the following anti-human antibodies: CD122 (BB515), CD127 (BB700), CD3 (BUV395), CD56 (BUV563), HLADR (BUV661), CD27 (BUV737), CD45 (BUV805), CD123 (AlexaFluor647), CD19 (APC-R700), CD14 (APCCy7), CD135 (PE), KLRG1 (PEDazzle594), CD10 (PeCy5), CD90 (PeCy7), CD66b (BV421), CD7 (BV480), CD16 (BV510), CD8 (BV570), IgM (BV570), CD38 (BV605), CD45Ra (BV650), IgD (BV711), CD4 (BV750), CD34 (BV786). The antibody mixes also included the viability dye LIVE/DEAD™ Fixable Blue (Life Technologies) and anti-mouse CD45.1 (APCCy7). Flow cytometry was performed on a BD FACSymphony (BD Biosciences). Fluorophore compensations and detector voltage were set up using single stains on human PBMCs and murine spleen cells. See Additional file 3: Fig. S3 for gating strategy. Human PBMCs from healthy donors were collected in accordance with CIRB Ref 2017/2806. Data was recompensated and analyzed using FlowJo v10 software (FlowJo LLC).

### Single cell RNA sequencing

Spleens were harvested from three young (14-week-old) and three aging (55-week-old) humanized mice and dissociated by mashing through a 100 μm cell strainer to obtain single cells. RBC were removed using ACK lysis buffer (Gibco). Human immune cells were purified using dead cell removal kit (Miltenyi Biotec) and mouse cell depletion kit (Miltenyi Biotec), pooled into two samples (“Young” and “Aging”), before being processed individually on the Chromium Controller (10 × Genomics). Libraries were prepared using the Chromium Next GEM Single Cell 3' Kit v3.1 (10 × Genomics), pooled, and sequenced on the NovaSeq 6000 S4 (Illumina). Data processing and analysis were performed using Partek® Flow® (Partek).

### In-vitro stimulation and analysis of T cells

Isolated splenocytes were seeded into 96-well plates and stimulated for 4 h with 10 µg/mL PMA (Sigma-Aldrich) and 1 µg/mL Ionomycin (Sigma-Aldrich). Brefeldin A (Life Technologies) and Monensin (Life Technologies) were added 1 h after the start of stimulation. For flow cytometry, cells were stained with antibodies against CD3 (BUV395), CD27 BUV737), CD45 (BUV805), CD57 (AlexaFluor647), CD154 (PE-Vio 770), CD8 (BV570), CD45Ra (BV650), IFN-γ (APCCy7), TNF-α (PE), Granzyme B (PECF594), Perforin (Pacific Blue), CD4 (BV750) after 3 h. Alternatively, T cell stimulation was performed by coating a 96-well flat bottom plate with PBS-diluted anti-CD3 (Purified, eBioscience) (5 µg/mL) and incubating at 37 °C for 4 h. Diluted anti-CD28 (Purified, BD Biosciences) (5 µg/mL) with isolated splenocytes were added after removal of unbound anti-CD3 antibodies. Supernatants were harvested for LEGENDplex™ (Biolegend) analysis (see below) after 72 h incubation at 37 °C.

### Tumor development study

Liver cancer cell line PLC/PRF/5 (1 × 10^6^ cells) was injected subcutaneously into the flank of 18- and 60-week-old humanized mice, and tumor growth was monitored weekly by caliper measurement to derive the volume using the formula $\frac{\pi }{6} \left(length \times {width}^{2}\right).$ Four weeks post engraftment, tumors were harvested and dissociated using the human Tumor Dissociation Kit (Miltenyi Biotec) with the gentleMACS™ Octo Dissociator (Miltenyi Biotec). Cell suspension was layered over 35% Percoll® (GE Healthcare) and centrifuged to purify the tumor infiltrating lymphocytes (TILs) for downstream flow cytometry analysis. Antibodies used include anti-human CD4 (SK3, BD Biosciences), anti-human KLRG1 (14C2A07, Biolegend), anti-human CD3 (HIT3a, BD Biosciences), anti-human CD45RA (HI100, Biolegend), anti-human CTLA-4 (BN13, Biolegend), anti-human CD45 (H130, BD Biosciences), anti-human PD1 (EH12.2H7, Biolegend), anti-human LAG3 (11C3C65, Biolegend), anti-human CD27 (O323, Biolegend), anti-human CD8 (SK1, BD Biosciences), anti-human TIGIT (MBSA43, ebioscience), and anti-human TIM3 (F38-2E2, Biolegend). Data was acquired on a LSR II flow cytometer (BD Biosciences) and analyzed using the FlowJo software v10 (FlowJo LLC). Exclusion criteria include humane endpoints as stated in the IACUC protocol, such as maximum tumor size, weight loss and animal constitution.

### Quantification of multiple immune analytes (cytokines, chemokines, proteases)

Immune analytes from blood plasma and cell culture supernatant were quantified using pre-defined LEGENDplex™ human panels (Biolegend) – Inflammation Panel 1, and CD8/NK Panel and T Helper Cytokine Panel V2, respectively, according to the manufacturer’s instructions. Data was acquired on a LSR II flow cytometer (BD Biosciences) and analyzed using the LEGENDplex™ Data Analysis Software Suite. Collection of blood plasma was via submandibular bleeding followed by centrifugation.

### Statistical analysis

Prism 9 software (GraphPad) was used to perform statistical analysis. Individual mouse constitutes an experimental unit. Power calculation was not performed and at least three biological replicates (as indicated) were used to determine statistical significance. Unless stated, data are represented as mean ± SD and were analyzed using Mann–Whitney U test. *P* value ≤ 0.05 denotes statistical significance.

### Supplementary Information

**Additional file 1:** **Figure S1.** Percentages of human reconstitution and immune subsets. Raw data for Fig. 1.

**Additional file 2:** **Figure S2.** Percentages of human reconstitution and immune subsets. (A to D) Proportion of naïve CD4^+^ and CD8^+^ T cell subsets in blood (A), lung (B), spleen (C) and liver (D). Kruskal-Wallis test was used for significance tests. Box and whisker plots are shown and p values are indicated; *n* = 5.

**Additional file 3:** **Figure S3.** FACS gating strategy. Gating to distinguish the indicated immune cells. The sample represented is blood tissue from a week 16.5 mouse.

**Additional file 4:** **Figure S4.** Single cell RNA sequencing analysis of splenocytes. tSNE plot showing nine graph-based clusters highlighting the ones that represent the indicated immune subset. Table shows top ten expressed genes for each of the nine clusters and the immune cell type (including the immune cell expression cluster in parenthesis) associated with each gene (according to The Human Protein Atlas).

## Data Availability

Data generated and analyzed during this study are included in this published article and its supplementary information files. Other datasets are available from the corresponding author on reasonable request.

## Abbreviations

CTLA4: Cytotoxic T-lymphocyte-associated protein 4
DN: Double negative
DP: Double positive
FBS: Fetal bovine serum
GM-CSF: Granulocyte–macrophage colony-stimulating factor
GO: Gene ontology
GSEA: Gene set enrichment analysis
HSC: Hematopoietic stem cell
ICS: Intracellular staining
IFNγ: Interferon-gamma
IL: Interleukin
KLRG1: Killer cell lectin like receptor G1
LAG3: Lymphocyte activation gene 3
M-CSF: Macrophage colony-stimulating factor
MHC: Major histocompatibility complex
NCAM1: Neural cell adhesion molecule 1
NIKO: NOD-*scid IL2rγ*^*−*^^*/*^^*−*^
NK: Natural killer
NOD: Non-obese diabetic
PBS: Phosphate buffered saline
PD1: Programmed cell death protein 1
RBC: Red blood cell
RNA: Ribonucleic acid
SCID: Severe combined immunodeficient
SD: Standard deviation
TIGIT: T cell immunoreceptor with Ig and ITIM domains
TIM3: T-cell immunoglobin domain and mucin domain protein 3
TIL: Tumor infiltrating lymphocyte
TNF: Tumor necrosis factor
Treg: Regulatory T cell
tSNE: T-distributed stochastic neighbor embedding
