# Supplementary material for: Towards a better preclinical cancer model – human immune aging in humanized mice
Source: Immun Ageing. 2023 Sep 27;20:49. doi: 10.1186/s12979-023-00374-4 (PMC10523735; doi:10.1186/s12979-023-00374-4)
Supplement: Supplementary file 1 — Additional file 1: Figure S1. Percentages of human reconstitution and immune subsets. Raw data for Fig. 1. [file 12979_2023_374_MOESM1_ESM.pdf]

Tan et al. Figure S1

A

Organ % reconstitution	Blood	BM	Liver	Lung	Spleen	Thymus
Week 1	2.60	2.49	11.68	5.58	2.85	NA
Week 3	6.64	9.80	19.26	4.65	3.10	81.41
Week 6	9.71	29.23	66.17	15.52	29.74	96.18
Week 9	22.91	47.55	83.01	40.45	68.46	98.67
Week 16.5	37.54	56.83	87.17	50.95	77.55	99.80
Week 18	72.19	NA	NA	NA	73.59	NA
Week 28.5	47.67	44.12	90.07	64.94	72.55	96.43
Week 36	14.32	23.89	60.97	31.84	48.78	80.07
Week 44	38.89	38.20	86.86	63.97	54.50	97.28
Week 52	22.00	24.06	82.92	45.38	42.45	66.77

B

Organ	Week	CD3	B cells	NK	Myeloid	Gran	CD34
Blood	1	0.15%	2.19%	2.86%	29.27%	7.43%	6.57%
Blood	3	0.00%	28.14%	6.49%	10.90%	5.75%	0.78%
Blood	6	0.19%	69.29%	0.77%	19.30%	0.51%	0.87%
Blood	9	11.23%	65.83%	0.95%	14.44%	0.25%	0.35%
Blood	16.5	27.12%	58.58%	1.08%	8.30%	0.50%	0.21%
Blood	18	70.88%	6.83%	2.07%	15.81%	0.81%	0.07%
Blood	28.5	97.06%	1.36%	0.51%	0.11%	0.02%	0.01%
Blood	36	76.28%	9.12%	0.60%	11.02%	0.72%	0.08%
Blood	44	84.74%	1.99%	1.09%	9.50%	0.79%	0.36%
Blood	52	83.29%	2.88%	0.72%	10.06%	0.35%	0.07%

Organ	Week	CD3	B cells	NK	Myeloid	Gran	CD34
Lung	1	0.04%	3.83%	0.25%	70.19%	3.10%	4.21%
Lung	3	0.04%	24.84%	0.92%	62.35%	0.32%	0.61%
Lung	6	0.18%	40.59%	0.77%	47.89%	0.63%	0.70%
Lung	9	12.10%	54.56%	1.23%	25.68%	0.28%	0.21%
Lung	16.5	34.39%	35.89%	1.56%	22.59%	0.91%	0.23%
Lung	28.5	93.42%	2.05%	0.66%	0.39%	0.53%	0.01%
Lung	36	70.68%	5.04%	1.06%	16.99%	2.32%	0.07%
Lung	44	82.49%	1.76%	1.75%	7.12%	3.06%	0.06%
Lung	52	74.34%	2.26%	1.33%	12.39%	1.41%	0.27%

Organ	Week	CD3	B cells	NK	Myeloid	Gran	CD34
Liver	1	0.03%	5.98%	0.17%	75.21%	2.67%	2.62%
Liver	3	0.02%	55.23%	0.66%	34.32%	1.88%	0.50%
Liver	6	0.09%	38.96%	0.60%	47.27%	0.91%	1.51%
Liver	9	21.01%	46.68%	0.71%	23.73%	0.53%	0.79%
Liver	16.5	43.97%	38.83%	1.20%	10.63%	1.21%	0.44%
Liver	28.5	96.67%	0.88%	0.39%	0.10%	0.32%	0.01%
Liver	36	78.58%	7.76%	0.59%	8.50%	2.45%	0.29%
Liver	44	88.68%	0.96%	1.60%	3.53%	2.93%	0.03%
Liver	52	83.16%	1.64%	1.02%	6.95%	5.46%	0.16%

Organ	Week	CD3	B cells	NK	Myeloid	Gran	CD34
Spleen	1	0.16%	8.08%	0.12%	59.22%	8.00%	5.77%
Spleen	3	0.36%	55.94%	0.80%	16.47%	1.75%	0.55%
Spleen	6	0.06%	84.99%	0.58%	7.23%	0.39%	0.74%
Spleen	9	4.97%	87.88%	0.39%	2.52%	0.19%	0.19%
Spleen	16.5	17.64%	73.87%	1.24%	2.26%	1.06%	0.26%
Spleen	18	78.87%	34.46%	0.92%	3.90%	0.54%	0.05%
Spleen	28.5	66.08%	15.52%	0.46%	0.22%	1.09%	0.02%
Spleen	36	75.50%	24.52%	0.44%	1.07%	4.37%	0.15%
Spleen	44	74.96%	12.87%	1.18%	1.33%	2.97%	0.05%
Spleen	52	60.13%	13.07%	0.87%	1.69%	4.77%	0.12%

Organ	Week	CD3	B cells	NK	Myeloid	Gran	CD34
BM	1	0.05%	3.45%	4.94%	59.76%	0.40%	17.64%
BM	3	0.02%	86.14%	0.23%	8.02%	0.13%	1.57%
BM	6	0.02%	86.18%	0.21%	8.38%	1.08%	2.16%
BM	9	0.58%	88.89%	0.11%	5.85%	0.39%	1.76%
BM	16.5	3.71%	85.30%	0.16%	6.12%	1.02%	1.25%
BM	28.5	89.44%	2.14%	0.28%	2.67%	0.00%	0.67%
BM	36	39.13%	37.44%	0.25%	7.10%	0.45%	0.54%
BM	44	61.54%	8.75%	0.56%	8.76%	0.04%	0.58%
BM	52	58.07%	3.88%	0.70%	10.86%	0.03%	0.47%

Organ	Week	DN	DP	CD4	CD8	B cells	NC T cells
Thymus	3	18.13%	13.97%	13.94%	0.08%	41.02%	2.159775
Thymus	6	5.36%	58.46%	6.25%	0.74%	20.29%	0.419886
Thymus	9	3.31%	43.04%	23.81%	14.97%	7.89%	0.416353
Thymus	16.5	2.27%	74.05%	10.50%	2.16%	5.63%	0.621381
Thymus	28.5	1.64%	35.33%	41.11%	17.74%	0.33%	0.592796
Thymus	36	8.55%	19.98%	45.92%	11.19%	4.76%	1.639158
Thymus	44	2.28%	27.03%	40.92%	19.36%	3.31%	1.048336
Thymus	52	6.54%	38.25%	35.19%	9.29%	3.25%	0.764051
Thymus	60	5.34%	19.40%	40.83%	21.90%	1.18%	1.272891
