# Supplementary figures and images for: Towards a better preclinical cancer model – human immune aging in humanized mice
Source: Immun Ageing. 2023 Sep 27;20:49. doi: 10.1186/s12979-023-00374-4 (PMC10523735; doi:10.1186/s12979-023-00374-4)

**A**

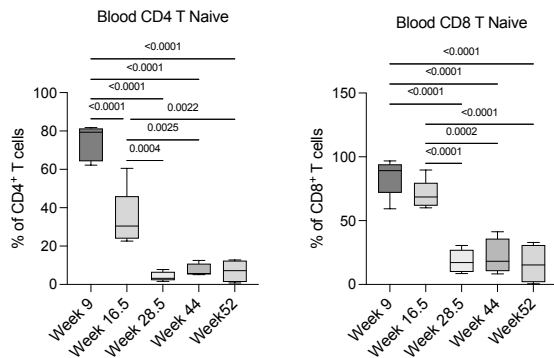

**B**

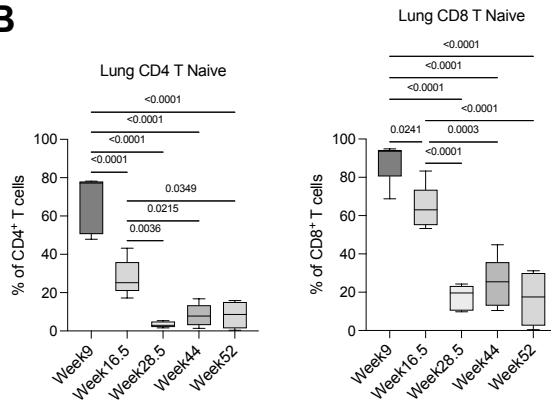

**C**

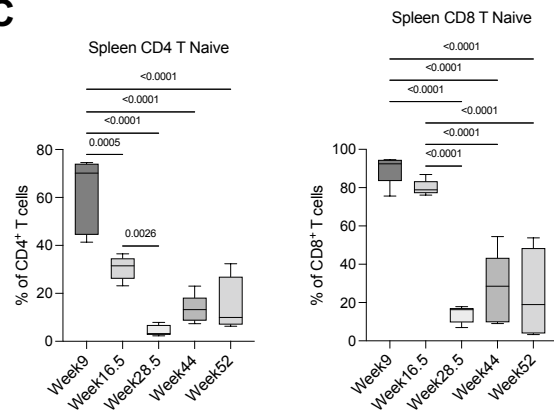

**D**

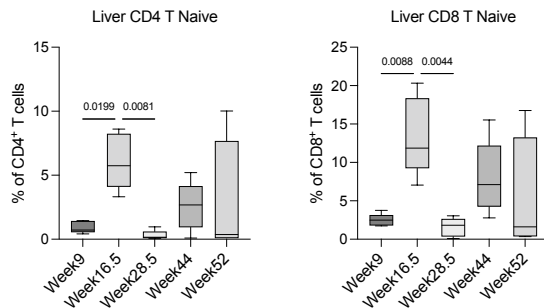

Supplement: Supplementary file 2 — Additional file 2: Figure S2. Percentages of human reconstitution and immune subsets. (A to D) Proportion of naïve CD4+ and CD8+ T cell subsets in blood (A), lung (B), spleen (C) and liver (D). Kruskal-Wallis test was used for significance tests. Box and whisker plots are shown and p values are indicated; n = 5. [file 12979_2023_374_MOESM2_ESM.pdf]
