# Supplementary material for: Towards a better preclinical cancer model – human immune aging in humanized mice
Source: Immun Ageing. 2023 Sep 27;20:49. doi: 10.1186/s12979-023-00374-4 (PMC10523735; doi:10.1186/s12979-023-00374-4)

Tan et al. Figure S3

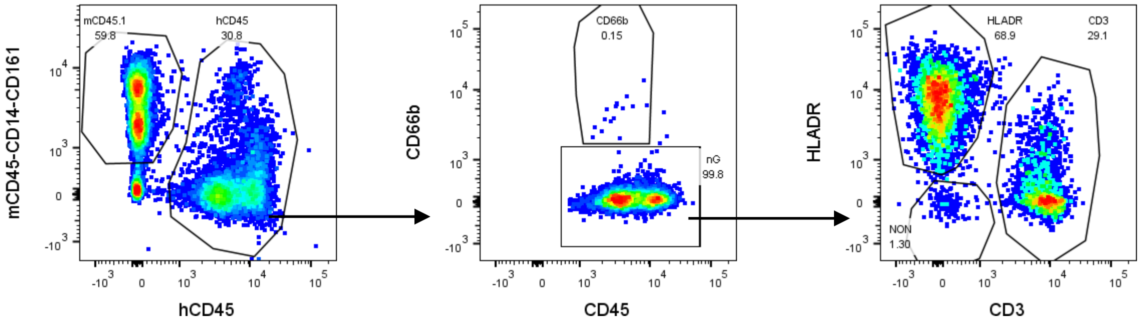

CD3^{+} non-conventional T cells and CD4^{+} CD8^{+} T cells

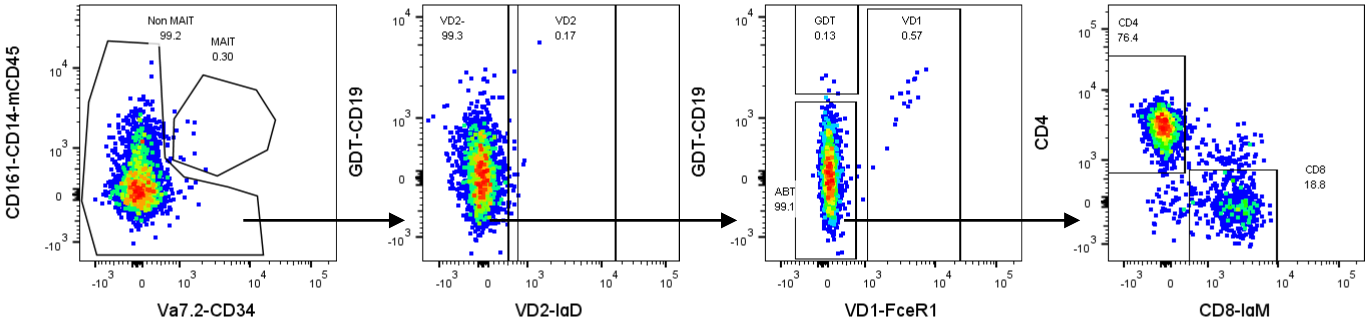

CD3^{+} memory, senescent and exhausted subsets

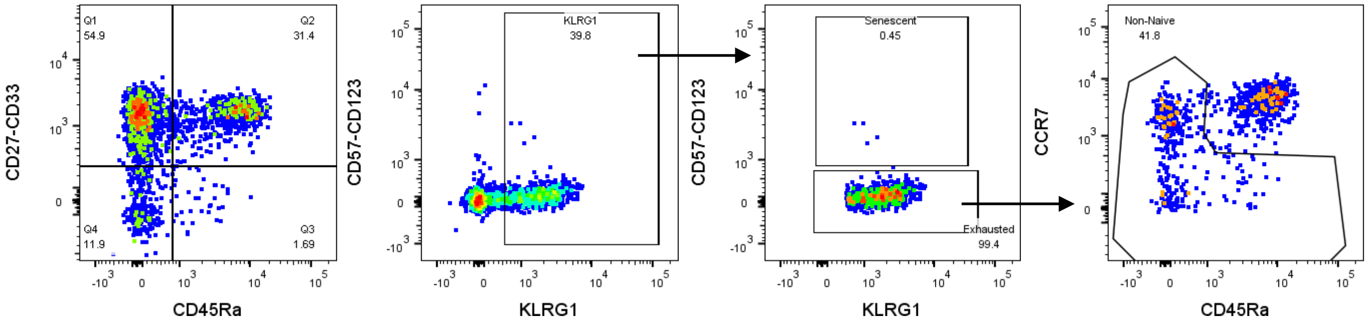

CD3^{-} HLADR^{+} cells

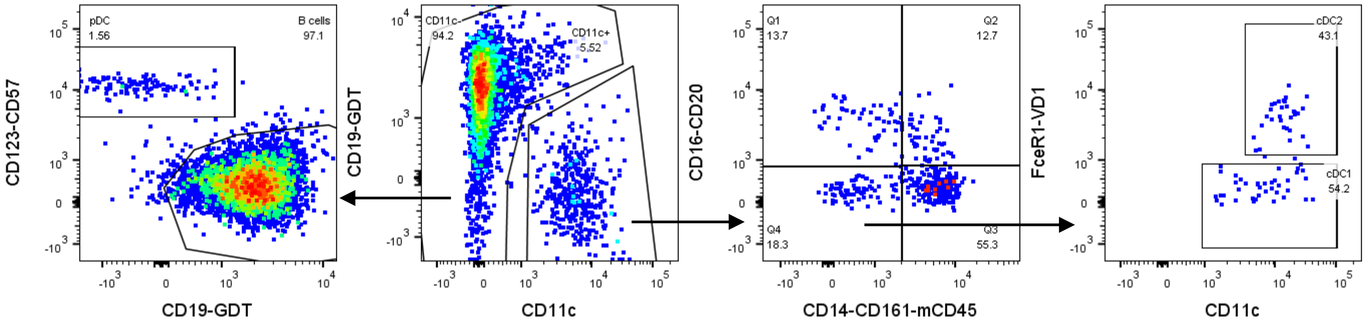

CD3^{-} HLADR^{-} cells

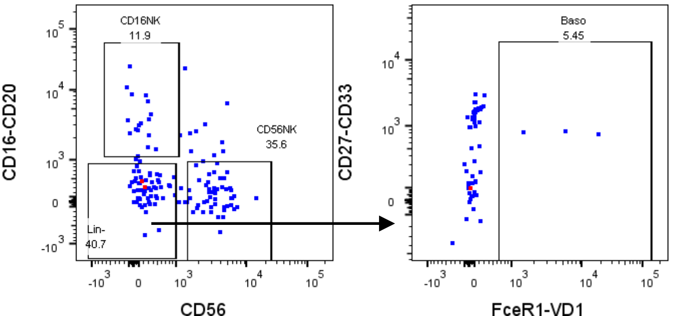

Supplement: Supplementary file 3 — Additional file 3: Figure S3. FACS gating strategy. Gating to distinguish the indicated immune cells. The sample represented is blood tissue from a week 16.5 mouse. [file 12979_2023_374_MOESM3_ESM.pdf]
