# Supplementary material for: Towards a better preclinical cancer model – human immune aging in humanized mice
Source: Immun Ageing. 2023 Sep 27;20:49. doi: 10.1186/s12979-023-00374-4 (PMC10523735; doi:10.1186/s12979-023-00374-4)
Supplement: Supplementary file 4 — Additional file 4: Figure S4. Single cell RNA sequencing analysis of splenocytes. tSNE plot showing nine graph-based clusters highlighting the ones that represent the indicated immune subset. Table shows top ten expressed genes for each of the nine clusters and the immune cell type (including the immune cell expression cluster in parenthesis) associated with each gene (according to The Human Protein Atlas). [file 12979_2023_374_MOESM4_ESM.pdf]

Tan et al. Figure S4

CD3$^+$ T cells

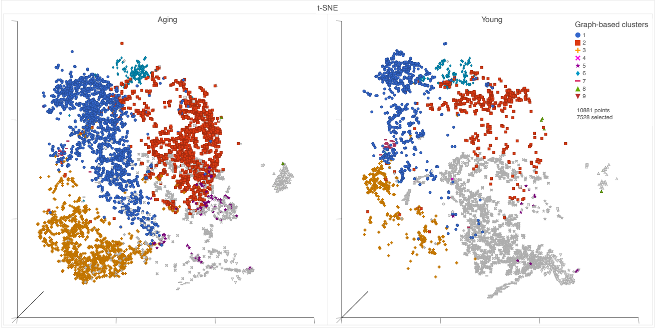

CD8$^+$ (CD4$^-$) T cells

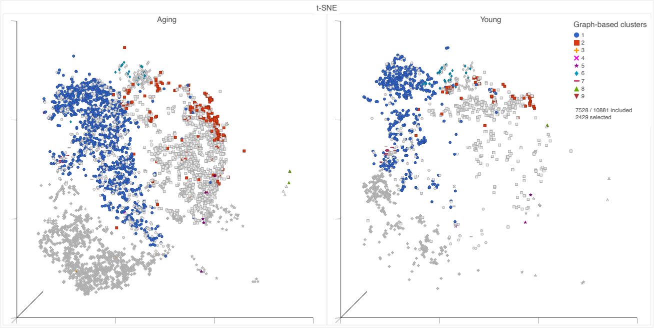

CD4$^+$ (CD8$^-$) T cells

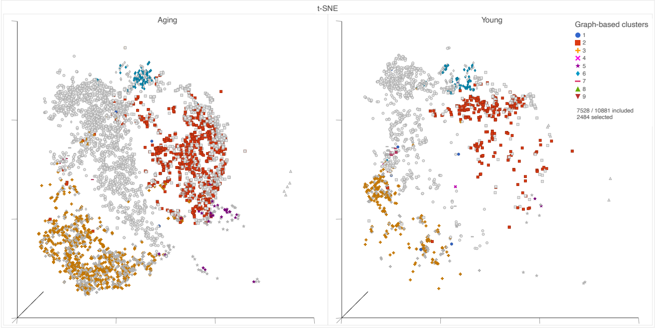

B cells

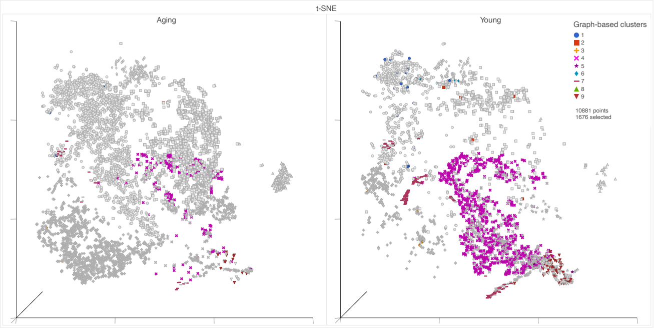

NK cells

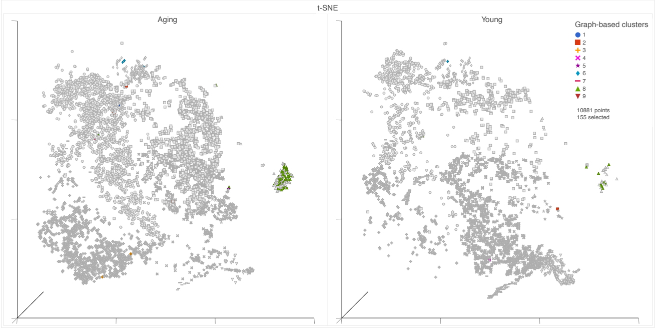

KLRG1$^+$ cells

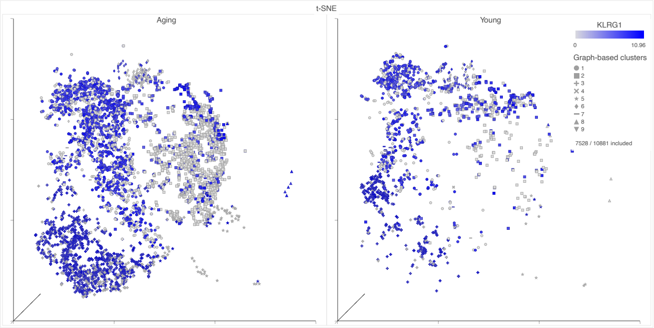

Cluster 1 (CD8$^+$ T cells)		Cluster 2 (CD4 / ⊆CD8 T cells)		Cluster 3 (CD4$^+$ T cells)		Cluster 5 (Granulocytes - Neutrophils)		Cluster 6 (Tregs)	
CD248	T cells (32)	LINC00892		NRP2		RPL23	Non-specific - Translation (30)	H1-5	
**CD8B**	T cells (32)	TSHZ2	T cells - TCR (20)	FAM13A	Monocytes (7)	MTRNR2L10	Neutrophils - Mixed function (1)	RRM2	GdT cells - Adaptive immune response (36)
CPNE2	Eosinophils - Transcription (52)	TNFRSF4	Tregs (35)	CD40LG	T cells (32)	MTRNR2L6	Neutrophils - Mixed function (1)	UBE2C	Tregs (35)
LINC02446		PDCD1	Immune cells - Immune response (16)	IL6ST	T cells (32)	SERBP1		GTSE1	Eosinophils - Innate immune response (45)
IFNG-AS1		CCR4	Tregs - Cell cycle regulation (4)	FHIT	T cells (32)	CTSG	Neutrophils - Chromatin organization (33)	CEP55	Tregs - Cell cycle regulation (4)
GZMK	MAIT* (16)	LGALS1	Monocytes - Inflammatory response (25)	KLRG1	MAIT* (16)	CDH11		DLGAP5	Tregs - Cell cycle regulation (4)
STAG3		CYTOR		SUSD4	T cells (32)	CPA3	Basophils (38)	CDK1	NK cells - Transcription (15)
**CD8A**	GdT cells - Adaptive immune response (36)	LGALS3	Monocytes - Inflammatory response (25)	**CD4**	Plasmacytoid DCs (3)	SOX6	Neutrophils - Mixed function (1)	ASPM	
KLRC4	GdT cells - Adaptive immune response (36)	MIR4435-2HG		TCF7	T cells (32)	AL157895.2		PCLAF	Tregs - Cell cycle regulation (4)
NELL2	T cells (32)	MAF	Basophils (38)	TIMP1	Monocytes (7)	RHEX	Basophils (38)	BIRC5	Tregs - Cell cycle regulation (4)

Cluster 4 (B cells)		Cluster 7 (B cells)		Cluster 9 (B cells)		Cluster 8 (NK cells)	
GNG3	Naïve and Memory B cells	IGLL1	B cells (24)	PCDH9	B cells - Adaptive immune response (21)	KLRF1	NK cells
PNOC	B cells - Humoral Immune response (19)	RAG2		NIBAN3	Plasmacytoid DCs (3)	FGFBP2	GdT cells - Adaptive immune response (36)
PPP1R14A	B cells - Transcription (37)	ARPP21	Plasmacytoid DCs - Protein folding (47)	FCRL5	Naïve and Memory B cells	KIR2DL4	NK cells (48)
IGHD	B cells - Adaptive immune response (21)	VPREB1	B cells - Transcription (37)	RALGPS2	B cells - Humoral Immune response (43)	XCL2	NK cells (48)
BANK1	B cells - Humoral Immune response (19)	AKAP12	Basophil	MS4A1	B cells - Humoral Immune response (43)	SPTSSB	NK cells (48)
FCER2	B cells - Adaptive immune response (21)	CFAP73		IGHM	B cells - Adaptive immune response (21)	XCL1	NK cells (48)
MS4A1	B cells - Humoral Immune response (43)	P4HA2	Neutrophils - Mixed function (1)	FCRL1	B cells - Adaptive immune response (21)	IGFBP7	Eosinophils - Transcription (52)
LINC01781		TNFRSF17	Naïve and Memory B cells, Plasmacytoid DCs	MALAT1		**NCAM1**	NK cells (48)
GAPT	Eosinophils - Innate immune response (45)	MME	Neutrophils - Innate immune response (9)	CIITA	APC - Antigen presentation (23)	GNLY	NK cells (48)
ARHGAP24	B cells - Transcription (37)	IRF4	Naïve and Memory B cells, DCs, Treg	BCL11A	Plasmacytoid DCs (3)	KLRC1	NK cells (48)
